# Development and psychometric properties of a questionnaire on adolescents’ home food environment

**DOI:** 10.1017/S1368980025100402

**Published:** 2025-06-04

**Authors:** Camila Batista Rodrigues, Emanuele Souza Marques, Rosangela Alves Pereira

**Affiliations:** 1 Graduate Program in Nutrition, Federal University of Rio de Janeiro, Rio de Janeiro, Brazil; 2 Institute of Social Medicine, Rio de Janeiro State University, Rio de Janeiro, Brazil; 3 Department of Social and Applied Nutrition, Federal University of Rio de Janeiro, Rio de Janeiro, Brazil

**Keywords:** Home environment, Adolescent, Food, Surveys and questionnaires, Validation study

## Abstract

**Objective:**

To describe the design and psychometric assessment of the Adolescent Home Food Environment Questionnaire (Acronym in Portuguese: QAAD).

**Design:**

This was a cross-sectional study. Data were collected between August 2021 and January 2022 through self-administered questionnaires via a survey management application accessible by computer or smartphone. The instrument was subjected to analysis by a panel of experts and to a pretest that enabled the adjustment of the language and the reformulation of the questions. The psychometric evaluation included the assessment of test–retest reliability (intraclass correlation coefficient), internal consistency (composite reliability), structural validity (exploratory structural equation modelling and confirmatory factor analysis) and construct validity (Mann‒Whitney test; *P*< 0·05). The following food environment aspects were evaluated: family eating practices, food availability and accessibility, cooking equipment availability and parental feeding style. Moreover, the weekly frequency of fruit, bean and added sugar beverage consumption was assessed.

**Setting:**

A public high school in Rio de Janeiro, Brazil.

**Participants:**

14-to-19-year-old students (*n* 34 in the test‒retest reliability study; *n* 501 in the validation analysis).

**Results:**

The final version of the QAAD included thirty-two questions allowing the assessment of seven dimensions of the home food environment. The QAAD demonstrated satisfactory reliability (ICC ranging from 0·44 to 0·78), adequate internal consistency (composite reliability > 0·70) and satisfactory structural and construct validity.

**Conclusions:**

The careful QAAD design provided a valid, reliable and consistent instrument for characterising adolescents’ home food environments, which may provide information for tailoring and targeting healthy eating promotion actions aimed at adolescents.

The assessment of the food environment, defined as the interface between food systems and diets, has gained increased interest from researchers and health organisations due to its influence on food choice and consumption and, consequently, health outcomes^(1–3)^.

The diet of Brazilian adolescents is characterised by the consumption of energy-dense foods with excessive fat, sugar and Na contents, especially ultra-processed foods, combined with a reduced intake of fresh foods, such as fruits and vegetables^(4,5)^. Furthermore, Brazilian adolescents often adopt eating habits that are associated with unfavourable health outcomes, such as skipping breakfast^(6–8)^ and replacing full meals with low-nutritional-quality snacks^(9)^. In Brazil, the most commonly consumed foods by adolescents in the 2017–2018 National Dietary Survey were rice, beans, coffee and bread^(10)^. In addition, 75 % of the adolescents reported having three main meals (breakfast, lunch and dinner), and 88 % reported consuming at least one snack throughout the day^(11)^. Rice and beans, the main staple foods in Brazil, are usually eaten at lunch and dinner, whereas bread and coffee are common breakfast items. Food choices and meal habits are addressed in the Brazilian dietary guidelines, which recommend a diet based on fresh foods beyond encouraging regular consumption of breakfast, lunch and dinner, preferably shared with family or friends, habits that are related to healthier food choices^(12)^.

Although adolescents are gaining autonomy and independence, most of their food consumption occurs at home^(13,14)^. Among Brazilian adolescents, 87 % of total energy intake is provided by food eaten at home^(10)^. Thus, the home food environment significantly influences adolescents’ eating habits. The home food environment is conceived as the place for the development, transmission and reproduction of eating habits and preferences. Nevertheless, its measurement is acknowledged as a complex challenge^(15,16)^, especially considering various dimensions, such as food availability, parents’ eating habits, family food practices^(17,18)^, family structure^(19)^, food security^(20)^ and food accessibility and convenience^(21)^. The scales used in the Eating and Activity over Time Project^(22)^ and the questionnaire proposed by Qiu *et al.* ^(23)^ are instruments that assess some dimensions of the home food environment. The former instrument, developed in the USA, addresses fruit and vegetable availability, parental support for healthy eating and family meal habits. The latter, developed in China, assesses parents’ practices regarding children’s eating habits and the availability of chips and sugar-added beverages.

Since the consumption of homemade meals is central to healthy eating promotion^(24)^, the presence of equipment that facilitates cooking and food storage, as well as food convenience and accessibility in the household^(21)^, is important in the assessment of the potential of the home food environment for providing healthy eating for adolescents. However, in general, instruments designed to assess adolescents’ food environment do not include these aspects.

Studies exploring adolescents’ perceptions of the home food environment are scarce, especially in Brazil^(22,23,25,26)^; however, according to Caspi *et al.* ^(27)^, eating behaviours and food accessibility are more appropriately assessed on the basis of individual perceptions. Therefore, a novel and comprehensive approach to evaluating adolescents’ food environments may be important for designing effective actions to promote healthy eating targeted at adolescents.

This study aimed to design and evaluate a multidimensional instrument to assess adolescents’ perceptions of the home food environment. An original questionnaire encompassing various dimensions and including aspects overlooked in existing instruments was designed on the basis of the literature on the subject^(14,15,28,29)^. Furthermore, in assessing the home food environment of Brazilian adolescents, the instruments must capture the particularities of eating habits and practices in Brazil. The proposed tool identifies critical aspects of the home food environment that influence adolescents’ eating habits, guiding initiatives and public policies to encourage family involvement in promoting healthy eating habits among Brazilian adolescents.

## Methods

This study was conducted to design and assess the reliability and validity of the Adolescents’ Home Food Environment Questionnaire (acronym in Portuguese: QAAD, standing for ‘*Questionário de Avaliação do Ambiente Alimentar Doméstico’*). The study design consisted of three stages: first, the questionnaire design; second, the reliability assessment and third, the validity assessment.

The study population comprised students enrolled on two campuses of a public high school in Rio de Janeiro, Brazil. As recommended by Terwee *et al.*, the minimum sample size for a satisfactory reliability study is thirty students^(30)^. The reliability assessment was conducted on a branch campus where 200 students (99 girls and 101 boys) were eligible. To assess instrument validity, the sample size was estimated considering a minimum of ten respondents per question, which allows for estimating the parameters needed to assess the psychometric properties of the scale^(31)^. As the instrument included forty-eight questions, a sample size of at least 480 students was estimated. The validity assessment was conducted on the main campus, where 1584 students (663 girls and 921 boys) were eligible. In both reliability and validity studies, all 14- to 19-year-old students enrolled in the selected high school campuses were eligible to participate in the study, and invitations were sent using the school’s official communication channels.

Data were collected during the period of social distancing due to the COVID-19 pandemic, when students carried out their academic duties remotely. The questionnaires were self-administered via a survey management application accessible by computer or smartphone. The links to access the questionnaires were sent by email to all eligible students. The reliability assessment took place in August 2021, and the validity assessment occurred between September 2021 and January 2022.

In the reliability study, students answered the QAAD twice, with a 2-week interval between responses. In the validation study, in addition to the QAAD, the students answered questions on demographic (gender, age, race) and socio-economic characteristics (education of the household head and participation in government assistance programs) and on the frequency of fruit, vegetable and sugar-sweetened beverage intake in the week prior to completing the questionnaire (later categorised as < 5 or ≥ 5 d per week). This categorisation was chosen to ensure coherence with publications on the frequency of consumption of healthy and unhealthy eating markers^(4,7)^ and facilitate comparisons with epidemiological studies and nutritional surveillance research conducted in Brazil.

### Home Food Environment Evaluation Questionnaire

The QAAD design was guided by a literature review conducted in 2021 across several databases (PubMed, SciELO, Google Scholar) and university repositories of theses and dissertations. The review utilised the following keywords: ‘food environment’, ‘home environment’, ‘adolescent’, ‘children’, ‘eating behaviour’, ‘food consumption’, ‘weight status’, ‘overweight’, ‘factors’ and ‘determinants’, along with the corresponding terms in Portuguese. Additionally, publications by government and international organisations focusing on food environments, adolescents’ food choices and childhood obesity were considered.

The literature review identified key dimensions deemed essential for inclusion in QAAD to evaluate the home food environment comprehensively. Furthermore, questionnaires such as those from the ‘Eating and Activity over Time Project – Project EAT’^(22)^, the ‘Family Life, Activity, Sun, Health and Eating – FLASHE study’^(32)^ and the ‘Nutrition Environment Measures Survey–Perceived’^(33)^ were used as references for the QAAD. The initial questionnaire consisted of forty-eight questions categorised into eight constructs: (a) family eating practices; (b) availability of fresh foods and whole grains; (c) availability of unhealthy foods; (d) availability of equipment for cooking and storing food; (e) accessibility of fruits and vegetables; and (f) motivational, (g) monitoring or controlling and (h) emotional behaviours of parents or guardians regarding adolescents’ food intake.

A panel of experts comprising nutritionists and school health researchers analysed the questionnaire, and a pretest was conducted with eleven adolescents aged 16–19 years who were not part of the study sample. These two steps facilitated language adjustments and enhanced the clarity of the instrument.

In the evaluation of family eating practices, adolescents were asked to rate their agreement with statements about the frequency of family meal practices, such as interactions during meals and the frequency of eating away from home or consuming takeaway meals over the 30 d preceding the questionnaire.

Yes/no questions assessed the availability of fresh foods (leafy vegetables, other vegetables, fruits, meat and beans), whole grains (oatmeal and brown rice), unhealthy foods (cookies, candies, desserts, sodas, sugar-sweetened beverages, processed meat, ready-to-eat meals) and cooking and food storage equipment (refrigerator and freezer, oven, microwave, air fryer, blender, orange squeezer, sandwich maker, pressure cooker).

To determine fruit and vegetable accessibility, adolescents were asked to express their agreement with statements regarding the accessibility of fruits and vegetables at home and the presence of a vegetable garden or fruit trees.

Parents’ or guardians’ motivational behaviours were assessed through questions about their consumption of vegetables, fruits and beans in the presence of the adolescents, as well as their encouragement for adolescents to consume these foods.

Parents’ or guardians’ monitoring or control behaviours were evaluated by inquiring whether they were concerned about adolescents’ eating habits, body weight and intake of fruits, vegetables, snacks, sodas and sugar-sweetened beverages. Adolescents were also asked if they felt pressured to eat meals even when they did not enjoy the food.

Parents’ or guardians’ emotional behaviours were assessed by asking if they offered food or drinks as rewards for good behaviour or to compensate for sadness, annoyance or irritation. Additionally, the adolescents were asked if alternatives were provided when they refused the food offered at a meal.

The QAAD assigns scores to each dimension, with higher scores indicating a home food environment more conducive to promoting healthy eating habits (see online supplementary material, Supplemental Material).

### Statistical analysis

The demographic and socio-economic profiles of the adolescents, along with their food consumption frequency, were described in terms of absolute and relative frequencies.

The evaluation of structural validity was conducted in three stages. Stage 1 involved a confirmatory factor analysis to evaluate the initial forty-eight-question eight-factor model. The model’s goodness-of-fit was assessed using the root mean square error of approximation < 0·06, the comparative fit index ≥ 0·90 and the Tucker‒Lewis Index ≥ 0·90^(34)^. The modification index and expected parameter changes were examined to identify potential anomalies in the model.

Given the inadequate fit and the anomalies identified in Stage 1, an exploratory approach (Stage 2) was initiated using principal component analysis, retaining factors with eigenvalues > 1·0, which was followed by exploratory structural equation modelling (ESEM), employing Geomin rotation^(34)^. The ESEM provides standard errors for all rotated parameters, enables overall model fit testing and examines the relationships between factor structures, contributing to a more robust evaluation of the latent structure. This approach integrates exploratory and confirmatory techniques within the framework of structural equation modelling and represents an advanced alternative to the traditional exploratory factor analysis in evaluating the structural validity of instruments such as the QAAD^(35,36)^.

The cross-loadings (high loadings on more than one factor) identified in the ESEM were analysed, and items were excluded based on the following sequential criteria: (1) the two highest factor loadings for a given item exceeded 0·45; (2) the lowest factor loading for a given item exceeded 0·35 and (3) the difference between the two highest loadings for a given item was less than 0·2. These criteria, which are based on the methodological framework outlined in Reichenheim *et al.* (2022)^(37)^, ensure that the retained items exhibit strong primary loadings (above the specified cut-off point) and minimal cross-loadings, thereby optimising the clarity and interpretability of the latent structure. After each round of ESEM and the subsequent elimination of items based on the criteria outlined above, a new ESEM iteration was performed until no item met the elimination criteria.

In Stage 3, a new confirmatory factor analysis was performed to validate the revised structure of the instrument, using the same goodness-of-fit criteria described earlier.

The reliability analysis was carried out solely on the final version of the QAAD. The intraclass correlation coefficient (ICC) was estimated to assess test‒retest reliability for each questionnaire dimension. The ICC values were categorised on the basis of Landis and Koch’s criteria: ≥ 0·81, almost perfect agreement; 0·61–0·80, substantial agreement; 0·41–0·60, moderate agreement; 0·21–0·40, fair agreement; 0·00–0·20, slight agreement and ≤ 0, poor agreement^(38)^. Internal consistency was evaluated by estimating the composite reliability, which is considered a more appropriate measure when confirmatory factor analysis is applied, with values > 0·70 deemed satisfactory^(31,39,40)^.

Construct validity was assessed by comparing the median scores for the home food environment dimensions across categories of selected food item consumption frequency (Mann‒Whitney test; *P*< 0·05). To complement the statistical significance test and assess the effect magnitude, Cohen’s *d* was estimated, with a significance level of *P*< 0·05 and a power of 0·80. Effect sizes are classified as small (0·20–0·49), moderate (0·50–0·79) or large (> 0·80)^(41)^. Statistical analyses were performed using SPSS version 19·0, Mplus version 8·7 (for factor analysis) and G * Power version 3.1.9.2, HHU (for Cohen’s d effect size estimation).

### Ethical issues

The study was approved by the Ethical Committee of Clementino Fraga Filho Hospital of the Federal University of Rio de Janeiro (HUCFF/UFRJ) under protocol 4.505.036. Authorisation from the administration of the participating educational institution was obtained. Informed consent was provided by the students and parental/guardian authorisation was required for the minors. During the test–retest phase, participants were identified by their email addresses to match responses over time. However, in the main phase of the study, all responses were anonymised to ensure that no personally identifiable information was collected.

## Results

In total, 501 students (32 % of eligible participants) responded to the QAAD in the validation stage. The sample’s mean age was 17 years (sd = 1·24); 51 % were female, 53 % were white, 51 % lived with both parents, 46 % reported that the mother/stepmother was the household head and 60 % of the household heads had ≤ 12 years of education. In the 12 months preceding the survey, 44 % of the families were assisted by a government assistance program. The consumption of fruits at least 5 days per week was reported by 28 % of the students, while 62 % reported eating beans and 17 % reported drinking sugar-sweetened beverages with the same frequency (Table 1).

**Table 1.** Characterisation of the students (*n* 501) participating in the adolescents’ home food environment questionnaire validation study, Rio de Janeiro, Brazil, 2021–2022

	*n*	%
Sex (*n* 501)		
Female	257	51
Male	244	49
Age (*n* 501)		
14–16 years	180	36
Over 16 years	321	64
Race/ethnicity (*n* 491)		
White	259	53
Brown	153	31
Black/Asian/Indigenous	79	16
Family structure (*n* 499)		
Living with both parents	256	51
Reconstituted family*	53	11
Single-parent family^†^	147	29
Others^‡^	43	9
Household head (*n* 487)		
Mother/stepmother	223	46
Father/stepfather	228	47
Others	36	7
Household head education (*n* 488)		
Up to 12 years	292	60
Over 12 years	196	40
Family assisted by a government assistance program (*n* 481)		
No	272	56
Yes	209	44
Fruit consumption frequency (*n* 499)		
< 5 d/week	359	72
≥ 5 d/week	140	28
Beans consumption frequency (*n* 500)		
< 5 d/week	191	38
≥ 5 d/week	309	62
Sugar-sweetened beverages consumption frequency^§^ (*n* 499)		
< 5 d/week	415	83
≥ 5 d/week	84	17

*n* = Absolute frequency;

* When father/mother marries again with other partners;

† refers to a single father/mother;

‡ grandfather/mother, uncle/ant, friend, alone;

§ sugar-sweetened beverages (iced tea, sugar-sweetened processed fruit drinks, drinks with guarana syrup, other).

In the initial stage of the structural validity analysis, the confirmatory factor analysis resulted in the indices root mean square error of approximation, comparative fit index and Tucker‒Lewis Index, which consistently indicated inadequate adjustment for the initial 48-question model. The factor loadings varied from 0·03 to 0·88, items with high residuals were observed (Table 2), and the Modification Index and the Standardised expected parameter changes exceeded the acceptable values. Cross-loadings across factors were also observed.

**Table 2.** Factor loadings, residuals and adjustments of the initial model and test–retest and composite reliability of the final model of the adolescents’ home food environment questionnaire. High school students (*n* 501), Rio de Janeiro, Brazil, 2021–2022

	Initial model (48 items)		Final model (32 items)				
	λ_1_	δ_*i*_	λ_2_	δ_*i*_	ICC^†^	95 % CI^‡^	CR^§^
Family eating practices					0·78^*^	0·57, 0·89	0·71
I1 In my family, we have at least one meal together a day	0·87	0·24	0·88	0·22			
I2 In my family, we really enjoy having meals together, but we can only do it on weekends	0·03	1·00	–	–			
I3 In my family, we meet and talk to each other during mealtimes	0·52	0·72	0·51	0·74			
I4 In my family, good manners are important during meals	0·57	0·68	0·54	0·71			
I5 In my family, it’s often difficult to find a time when everyone can have meal together	0·53	0·72	0·52	0·73			
I6 In my family, we watch TV and/or use smartphones or tablets during meals	0·41	0·83	–	–			
I7 I am usually too busy to have meals with my family	0·43	0·81	–	–			
I8 IN THE LAST 30 d, how many times has your family dined out or ordered bought products for delivery?	−0·24	0·94	–	–			
Fresh food and whole grain availability					0·72^*^	0·50, 0·85	0·80
I9 Oatmeal	0·54	0·70	0·53	0·71			
I10 Brown rice	0·17	0·97	–	–			
I11 Beans	0·63	0·60	0·54	0·71			
I12 Other vegetables	0·88	0·23	0·89	0·21			
I13 Leafy vegetables	0·67	0·54	0·69	0·52			
I14 Fruits	0·69	0·53	0·68	0·54			
I15 Meats	0·30	0·91	–	–			
Unhealthy food availability					0·77^*^	0·58, 0·88	0·72
I16 Cookies	0·57	0·67	0·63	0·60			
I17 Candies (including candy, chocolate, chewing gum, bonbon, lollipop)	0·59	0·65	0·57	0·68			
I18 Desserts	0·54	0·71	0·56	0·68			
I19 Sodas	0·54	0·71	–	–			
I20 Sugar-sweetened beverages	0·38	0·85	–	–			
I19 + I20 sodas and other sugar-sweetened beverages	–	–	0·51	0·74			
I21 Processed meats	0·55	0·70	0·49	0·76			
I22 Ready-to-eat meals	0·61	0·63	0·55	0·69			
Cooking equipment					0·72^*^	0·51, 0·85	0·75
I23 Refrigerator and freezer	0·46	0·79	–	–			
I24 Oven	0·82	0·32	0·60	0·64			
I25 Microwave	0·31	0·91	–	–			
I26 Air fryer	0·43	0·82	0·42	0·82			
I27 Blender	0·44	0·81	0·45	0·79			
I28 Orange squeezer	0·60	0·65	0·67	0·55			
I29 Sandwich maker	0·55	0·70	0·59	0·65			
I30 Pressure cooker	0·51	0·73	0·71	0·49			
Fruit and vegetable accessibility					0·73^*^	0·52, 0·86	0·85
I31 Fruits are stored in an easily accessible place	0·36	0·87	–	–			
I32 Fruits are ready for consumption (e.g., cleaned and sliced)	0·65	0·57	0·65	0·58			
I33 Leafy vegetables are ready for consumption (e.g., cleaned and chopped)	0·90	0·18	0·90	0·18			
I34 Other vegetables are ready for consumption	0·87	0·25	0·87	0·24			
I35 Individuals Garden or Community Garden or fruit trees	0·27	0·93	–	–			
Parents’ or guardians’ motivational behaviour					0·71^*^	0·48, 0·84	0·85
I36 My parents/guardians eat vegetables when I’m with them	0·87	0·24	0·88	0·23			
I37 My parents/guardians eat fruits when I’m with them	0·78	0·38	0·78	0·39			
I38 My parents/guardians eat beans when I’m with them	0·40	0·84	–	–			
I39 My parents/guardians encourage me to eat more fruits and/or vegetables	0·78	0·34	0·78	0·39			
Parents’ or guardians’ monitoring or controlling behaviours					0·44^*^	0·14, 0·67	0·85
I40 My parents/guardians care about my weight	0·55	0·70	0·55	0·70			
I41 My parents/guardians care about my diet	0·72	0·49	0·73	0·47			
I42 My parents/guardians check if I eat fruits and vegetables	0·79	0·37	0·79	0·38			
I43 My parents/guardians control my consumption of sugar-sweetened beverages	0·84	0·30	0·83	0·31			
I44 My parents/guardians control my snack consumption	0·76	0·42	0·76	0·41			
I45 My parents/guardians ensure that I eat that dish or food, even if I don’t like one of the dishes prepared for the family meal	−0·26	0·93	–	–			
Parents’ or guardians’ emotional behaviours							–
I46 My parents/guardians offer me food/drinks as a reward to convince me to complete tasks or follow orders or recommendations	0·61	0·63	–	–			
I47 My parents/guardians offer me foods/drinks when I’m sad, upset, or annoyed	0·45	0·79	–	–			
I48 When I don’t like the dishes prepared for the Family meal, I can choose another option that I prefer	0·12	0·99	–	–			
Model goodness-of-fit parameters:							
RMSEA	0·049(0·046–0·052)		0·045(0·040–0·049)				
CFI	0·779		0·914				
TLI	0·763		0·904				

RMSEA: root mean square error of approximation (< 0·06); CFI: comparative fit index (≥ 0·90); TLI: Tucker–Lewis Index (≥ 0·90).

λ_1_: Standardised factor loadings in the initial model; λ_2_: Standardised factor loadings in the final model; δ_*i*_: residuals.

* *P*< 0·05.

† ICC: intraclass correlation coefficient (test–retest reliability): ≥ 0·81, almost perfect; 0·61–0·80, substantial; 0·41–0·60, moderate; 0·21–0·40, fair; 0·00–0·20, slight and ≤ 0, poor agreement.

‡ 95 % CI.

§ Composite reliability: > 0·70 deemed satisfactory.

In the subsequent stage, ESEM indicated the need to exclude fifteen items with factor loadings below 0·30 and the convenience of merging the items ‘sodas’ and ‘sugar-sweetened beverages’ into a single question (‘sodas and other sugar-sweetened beverages’). In this case, the responses were combined as follows: the option ‘NO’ was considered if the respondent marked ‘no’ for both questions, and ‘YES’ was considered if the respondent selected ‘yes’ for at least one question. Model goodness-of-fit was considered satisfactory, and factor loadings > 0·30 were estimated for all items retained in the final model (Table 2).

Finally, the analyses yielded a parsimonious model composed of thirty-two items and seven dimensions. The subscale scores estimated for the final thirty-two-item QAAD were as follows: (a) family eating practices (4 questions): 0–16 points; (b) availability of fresh foods and whole grains (5 items): 0–5 points; (c) availability of unhealthy foods (6 items): 0–6 points; (d) cooking equipment availability (6 items): 0–6 points; (e) fruit and vegetable accessibility (3 questions): 0–12 points; (f) parents’ or guardians’ motivational behaviour (3 questions): 0–9 points and (g) parents’ or guardians’ monitoring or controlling behaviours (5 questions): 0–17 points. The scores for the dimensions *Family Eating Practices*, *Fruit and Vegetable Accessibility*, *Parents’ or Guardians’ Motivational Behavior*, and *Parents’ or Guardians’ Monitoring or Controlling Behaviors* increased as the degree of agreement with the statements or the frequency of certain behaviors increased. One point was awarded for the presence of fresh foods, whole grains or cooking equipment, while the presence of unhealthy foods was scored in reverse order. Therefore, for all dimensions, higher scores indicate a household food environment that more strongly supports adolescents’ healthy eating habits (see online supplementary material, Supplemental Table S1).

The test–retest reliability analysis was conducted with thirty-four students (17 % of the eligible students enrolled in the branch campus). Substantial agreement (ICC ranging from 0·61 to 0·80) was estimated for the following QAAD subscales: ‘family eating practices’ (ICC = 0·78), ‘fresh food and whole grain availability’ (ICC = 0·72), ‘unhealthy food availability’ (ICC = 0·77), ‘cooking equipment availability’ (ICC = 0·72), ‘fruit and vegetable accessibility’ (ICC = 0·73) and ‘parents’ or guardians’ motivational behaviour’ (ICC = 0·71), whereas moderate agreement was observed for the ‘parent’ or guardians’ monitoring or controlling behaviours’ dimension (ICC = 0·44) (*P*< 0·05 for all estimates). All the dimensions presented satisfactory internal consistency, with composite reliability values exceeding 0·70 (Table 2).

The construct validity of the QAAD was confirmed, as the median scores for the dimensions ‘availability of fresh foods and whole grains’, ‘cooking equipment availability’, ‘fruit and vegetable accessibility’ and ‘parents’ or guardians’ motivational behaviours’ were greater for adolescents consuming fruits ≥ 5 d/week than for those consuming fruits < 5 d/week. Compared with those consuming beans < 5 d/week, adolescents reporting bean consumption ≥ 5 d/week presented higher mean scores for ‘family eating practices,’ ‘fruit and vegetable accessibility,’ ‘parents’ or guardians’ motivational behaviours,’ and ‘parents’ or guardians’ monitoring or controlling behaviours’. Conversely, adolescents who drank sugar-sweetened beverages ≥ 5 d/week had lower mean scores for the unhealthy food availability dimension than those who consumed these beverages less frequently, as higher scores in this dimension indicate a lower availability of unhealthy foods. The Cohen’s d effect sizes ranged from small (> 0·2) to moderate (0·5–0·8); *P*< 0·05) (Table 3).

**Table 3:** Score^‡^ median (and interquartile range) for the adolescents’ home food environment questionnaire dimensions according to the frequency of consumption of fruits, beans and sugar-sweetened beverages. High school students (*n* 501), Rio de Janeiro, Brazil, 2021–2022

	Fruit frequency of consumption		Effect size^§^	Bean frequency of consumption		Effect size^§^	SSB frequency of consumption		Effect size^§^
	< 5 d/week	≥ 5 d/week		< 5 d/week	≥ 5 d/week		< 5 d/week	≥ 5 d/week	
Subscales (score range)	Score median IQR			Score median IQR			Score median IQR		
Family eating practices (0–16)	9·5(6·0–12·0)	10·0(5·0–12·0)	–	8·0(4·0–12·0)	10·0^*^(7·0–12·0)	0·3	10·0(6·0–12·0)	9·0(6·0–12·5)	–
Fresh food and whole grain availability (0–5)	4·0(4·0–5·0)	5·0^*^(4·0–5·0)	0·4	4·0(4·0–5·0)	5·0(4·0–5·0)	–	4·0(4·0–5·0)	5·0(4·0–5·0)	–
Unhealthy food availability (0–6)^†^	3·0(2·0–4·0)	3·0(2·0–4·0)	–	3·0(2·0–4·0)	3·0(2·0–4·0)	–	3·0(2·0–4·0)	2·0^*^(1·0–3·0)	0·2
Cooking equipment availability (0–6)	5·0(4·0–5·0)	5·0^*^(4·0–6·0)	0·2	5·0(4·0–6·0)	5·0(4·0–5·0)	–	5·0(4·0–5·0)	5·0(4·0–6·0)	–
Fruit and vegetable accessibility (0–12)	8·0(4·0–10·0)	9·5^*^(7·0–12·0)	0·5	8·0(4·0–10·0)	9·0^*^(5·0–11·0)	0·2	8·0(5·0–10·0)	8·0(5·0–10·0)	–
Parents’ or guardians’ motivational behavior (0–9)	6·0(5·0–7·0)	7·0^*^(6·0–9·0)	0·5	6·0(4·0–8·0)	7·0^*^(5·0–8·0)	0·3	6·0(5·0–8·0)	7·0(5·0–8·0)	–
Parents’ or guardians’ monitoring or controlling behaviors (0–17)	9·0(7·0–12·0)	10·0(7·0–13·0)	–	9·0(6·0–11·0)	10·0^*^(7·0–13·0)	0·4	10·0(7·0–13·0)	9·0(7·0–12·0)	–

IQR: Interquartile range SSB: Sugar-sweetened beverages (iced teas, sugar-sweetened processed fruit drinks, drinks with guarana syrup, other).

* *P*-value < 0·05 (Mann–Whitney test).

† Score for the dimension is reversed: ‘yes’ = 0, ‘no’ = 1.

‡ Higher scores indicate food environments more conducive to healthier choices.

§ The effect size quantifies the magnitude of the difference by indicating the relevance of the effect. Effect sizes were estimated only for items with a statistically significant difference between the categories of food frequency of consumption according to the Mann–Whitney test. The effect sizes are considered small (> 0·2), moderate (0·5–0·8) or large (> 0·8)^(41)^.

## Discussion

The evaluation of the QAAD, a tool designed to characterise adolescents’ home food environments, demonstrated that the instrument presents acceptable psychometric qualities, such as good test‒retest reliability, satisfactory internal consistency, consistent structural validity and adequate construct validity. The final version of the QAAD includes thirty-two questions across seven dimensions, encompassing the availability of healthy and unhealthy food items and cooking equipment and family eating behaviours and practices. The QAAD enables the estimation of scores to evaluate the home food environment’s potential to promote healthy eating among adolescents.

Instruments assessing adolescents’ perceptions of the home food environment are scarce, both internationally and in Brazil. In the United States, Nebling *et al.* ^(32)^ developed a questionnaire evaluating the home food environment, the community environment and the consumption of healthy and unhealthy foods. Similar to the present study, the questionnaire underwent expert scrutiny and usability tests, addressing parental norms and attitudes toward healthy eating, family meal habits and household food availability. Another comparable instrument is the Dietary and Lifestyle Questionnaire, developed in India by Rathi *et al.* ^(25)^. The authors evaluated adolescents’ perceptions of the home food environment across three dimensions − family food rules and food accessibility and availability at home − including questions about responsibility for meal preparation. Like this study, Rathi *et al.* reported moderate to almost perfect agreement for all items of the questionnaire. In the EAT project, Neumark *et al.* assessed the home food environment of American adolescents by addressing parental support for healthy eating, family food practices and fruit and vegetable availability. Similarly, the authors submitted the data to expert examination and assessed the test‒retest reliability, internal consistency and structural validity^(22)^. The QAAD builds upon and expands these instruments by incorporating multiple dimensions while undergoing extensive psychometric validation.

Overall, the test–retest reliability of the QAAD was considered acceptable^(38)^, although the estimated coefficient for ‘Parents’ or guardians’ Monitoring or Controlling Behaviours’ was lower than that for the other dimensions. Responses to questions in this dimension may involve emotions and perceptions about the adolescent–parent relationship, which could fluctuate over short periods. One limitation of test–retest reliability is the possibility of changes in traits of interest, such as attitudes and moods, between test administrations^(42)^. Thus, the moderate ICC estimated for this subscale likely does not significantly affect the overall reliability of QAAD.

The QAAD demonstrated satisfactory internal consistency across all seven dimensions. Although Cronbach’s alpha is more frequently used to analyse the internal consistency of instruments and scales, this study adopted composite reliability, as it is considered a more appropriate estimator when using confirmatory factor analysis^(31)^. Additionally, according to Rayock (1997), Cronbach’s alpha is sensitive to heterogeneity in item factor loadings, which may compromise reliability estimates^(39)^.

In addition to establishing structural validity through statistical analysis, the QAAD was also subjected to construct validity evaluation by comparing dimension scores across the categories of consumption frequency of markers of healthy and unhealthy eating. Consistent with findings from other studies, individuals with healthier eating habits reported more favourable home food environments for healthy eating. Effect sizes ranged from small to moderate, aligning with expectations for health-related variables, as a substantial portion of the variance in dependent variables may not be easily explained^(41)^.

The association of the home food environment with adolescents’ diet has been reported in studies conducted in Korea^(21)^ and in the United States^(43)^. For example, daily fruit consumption was greater among Korean adolescents with positive perceptions of fruit availability (0·71 *v*. 0·39 servings/day) and accessibility (0·70 *v*. 0·43 servings/day) at home than among those with negative perceptions^(21)^. Similarly, among American adolescents, the home availability of sugar-sweetened beverages was directly associated with the intake of these drinks (*ß* = 0·18, *P*< 0·001), with 16 % of the variance in beverage consumption explained by the family context^(43)^.

In addition to the physical aspects of the home food environment, this study identified associations between parental behaviours and family eating practices and the consumption of protective foods, such as fruits and beans. These findings align with those of Leme *et al.* ^(26)^, who reported that parental and caregiver support for healthy eating promotion was directly associated with fruit consumption in adolescent girls (*ß* = 0·26, *P*= 0·03).

### Limitations

A limitation of this study is the high proportion of nonresponses (68 %). The high nonresponse rate may be attributed to social distancing measures for COVID-19 prevention and control, as the research process was conducted remotely, including the invitation and recruitment of participants. However, the number of participants in the study is comparable to that in similar studies^(18,25,44)^, and the sample sizes exceeded the minimum required for both test−retest reliability and structural validation analysis.

Nevertheless, selection bias — particularly self-selection bias — cannot be ruled out, as students who responded to the questionnaire may differ from non-respondents in ways relevant to the study’s topics. Significant differences (χ2, *P*< 0·05) were found in the distribution of sex and age between respondents and all eligible students, the only variables available for the full eligible population. Among study participants, 49 % were male and 21 % were under 16 years old, compared to 58 % and 36 %, respectively, among eligible students. Given the sample’s limited representativeness, the results should be interpreted with caution.

### Implications for research and practice

Although adolescents gain greater autonomy in food choice, during this life stage, home meals remain the primary source of food^(13,14,44)^. Moreover, parental eating patterns, family food rules and home food availability appear to significantly influence adolescents’ eating behaviour^(45)^. The data obtained with the QAAD may provide valuable insights into these topics. During the COVID-19 pandemic, with remote schooling and social distancing measures, the home food environment became the predominant factor affecting adolescents’ food consumption.

Evaluating the home food environment and its potential impact on adolescents’ nutrition and health requires indicators that can distinguish the exposure gradient. However, instruments designed to evaluate adolescents’ perceptions of their home food environment are scarce^(46,47)^. The QAAD addresses this gap by employing robust development procedures, namely, a comprehensive review of the literature, expert perusal and psychometric testing. Furthermore, the QAAD integrates multiple dimensions to assess the potential of the home food environment for promoting healthy eating among adolescents, overcoming the limitations of instruments that focus on specific aspects. Finally, the QAAD benefits from being self-administered and available online, reducing costs and ensuring data quality control^(48,49)^.

The validated QAAD provides a reliable and consistent tool for assessing adolescents’ home food environments, offering valuable information for tailoring interventions aimed at healthy eating promotion targeting this population group.

## Supporting information

Rodrigues et al. supplementary material Rodrigues et al. supplementary material

## Supplementary material

For supplementary material accompanying this paper visit https://doi.org/10.1017/S1368980025100402
