# Supplementary material for: Development and psychometric properties of a questionnaire on adolescents’ home food environment
Source: Public Health Nutr. 2025 Jun 4;28(1):e107. doi: 10.1017/S1368980025100402 (PMC12264776; doi:10.1017/S1368980025100402)
Supplement: Rodrigues et al. supplementary material [file S1368980025100402sup001.pdf]

Supplementary Material

Table S1. Scores from the Adolescents' Home Food Environment Questionnaire (acronym in Portuguese: QAAD)

Items	Scores
Family Eating Practices	
In my Family...	
We have at least one meal together a day	I totally disagree = 0<br>I partially disagree = 1<br>I do not agree nor disagree = 2<br>I partially agree = 3<br>I totally agree = 4
We meet and talk to each other during mealtimes	I totally disagree = 0<br>I partially disagree = 1<br>I do not agree nor disagree = 2<br>I partially agree = 3<br>I totally agree = 4
Good manners are important during meals	I totally disagree = 0<br>I partially disagree = 1<br>I do not agree nor disagree = 2<br>I partially agree = 3<br>I totally agree = 4
It's often difficult to find a time when everyone can have meal together	I totally disagree = 4<br>I partially disagree = 3<br>I do not agree nor disagree = 2<br>I partially agree = 1<br>I totally agree = 0
Score	***0 -16***
Fresh Foods and Whole Grains Availability	
Oatmeal	Yes = 1
Beans	Yes = 1
Other Vegetables	Yes = 1
Leafy vegetables	Yes = 1
Fruits	Yes = 1
	0 - 5
Unhealthy Food Availability	
Cookies	No = 1
Candies	No = 1
Desserts	No = 1
Sodas and other sugar-sweetened beverages	No = 1
Processed meats	No = 1
Ready-to-eat meals	No = 1
Score	***0 - 6***
Equipment to Cook	
Oven	Yes = 1
Air fryer	Yes = 1

Items	Scores
Sandwich maker	Yes = 1
Blender	Yes = 1
Orange squeezer	Yes = 1
Pressure pan	Yes = 1
Score	***0 - 6***
Fruits and Vegetables Accessibility	
Fruits are ready for consumption (cleaned and sliced, when applicable)	I totally disagree = 0<br>I partially disagree = 1<br>I do not agree nor disagree = 2<br>I partially agree = 3<br>I totally agree = 4
Greens are ready for consumption (cleaned and chopped, when applicable)	I totally disagree = 0<br>I partially disagree = 1<br>I do not agree nor disagree = 2<br>I partially agree = 3<br>I totally agree = 4
Vegetables are ready for consumption (cooked)	I totally disagree = 0<br>I partially disagree = 1<br>I do not agree nor disagree = 2<br>I partially agree = 3<br>I totally agree = 4
Score	***0 - 12***
Motivational Behavior of Parents/guardians	
My parents/guardians...	
Eat vegetables when I'm with them	Never = 0<br>Sometimes = 1<br>Frequently = 2<br>Always = 3
Eat fruits when I'm with them	Never = 0<br>Sometimes = 1<br>Frequently = 2<br>Always = 3
Encourage me to eat more fruits and/or vegetables	Never = 0<br>Sometimes = 1<br>Frequently = 2<br>Always = 3
Score	***0 - 9***
Monitoring or Controlling Behaviors of Parents/guardians	
My parents/guardians...	
Care about my weight	I totally disagree = 0<br>I partially disagree = 1<br>I do not agree nor disagree = 2<br>I partially agree = 3<br>I totally agree = 4
Care about my diet	I totally disagree = 0<br>I partially disagree = 1<br>I do not agree nor disagree = 2<br>I partially agree = 3<br>I totally agree = 4

**Items**	**Scores**
Check if I eat fruits and vegetables	Never = 0<br>Sometimes = 1<br>Frequently = 2<br>Always = 3
Control my consumption of sugar-sweetened beverages	Never = 0<br>Sometimes = 1<br>Frequently = 2<br>Always = 3
Control my snack consumption	Never = 0<br>Sometimes = 1<br>Frequently = 2<br>Always = 3
Score	***0 a 17***
